# Moth oviposition shapes the species-specific transcriptional and phytohormonal response of *Nicotiana attenuata* to larval feeding

**DOI:** 10.1038/s41598-018-28233-z

**Published:** 2018-07-06

**Authors:** Sylvia Drok, Michele Bandoly, Sandra Stelzer, Tobias Lortzing, Anke Steppuhn

**Affiliations:** Freie Universität of Berlin/Institute of Biology/Dahlem Centre of Plant Sciences, Laboratory of Molecular Ecology, Albrecht-Thaer Weg 6, Berlin, 14195 Germany

## Abstract

Oviposition by lepidopteran herbivores on *Nicotiana attenuata* primes plant defence responses that are induced by the feeding larvae. While oviposition by both the generalist *Spodoptera exigua* and the specialist *Manduca sexta* primes the production of defensive phenylpropanoids, their larvae are differentially affected. We investigate here the impact of prior oviposition on the transcriptome and phytohormone levels of plants that were later attacked by larvae to find regulatory signals of this priming. In a full-factorial design, we evaluated the effects of oviposition and herbivory by both species. Oviposition alone had only subtle effects at the transcriptional level. Laval feeding alone induced species-specific plant responses. Larvae of the generalist regulated phytohormones and gene expression stronger than larvae of the specialist. A day after larvae started to feed, we detected no significant alterations of the plant’s response to larval feeding due to prior oviposition by conspecific moths. Yet, oviposition by each of the species profoundly influenced the plant’s transcriptional and phytohormonal response to feeding larvae of the other species. Remarkably, the species-specific plant responses to larval feeding shifted towards the response normally elicited by larvae of the ovipositing species. Thus, plants may already recognise an insect’s identity upon its oviposition.

## Introduction

Plants evolved a multitude of adaptations to resist herbivorous insects. In turn, insects evolved adaptations to find and colonise suitable host plants. Plants possess diverse defence traits which are often inducible upon herbivory^1^. Insects evolved traits to avoid, resist, or even manipulate a plant’s defence response^2^. The closer the evolutionary relationship between plants and insect herbivores, the more sophisticated and specialised defence and counter-defence can be geared to each other. The degree of such coevolution is driven by the insect’s impact on plant fitness and its host plant range. Specialised herbivores with narrow host plant ranges can often tolerate, detoxify or even co-opt plant defences for their own defence^3^. In several plants, generalist and specialist insects elicit different responses^4–6^, which may be adaptive for the plant or the insect^7^. Plants may render their defence responses most efficient against different insects that are differentially susceptible, and adapted insects may evolve traits to manipulate plant defence.

Inducible plant defences allow plants to limit their investments of resources into defence to circumstances in which these pay off, i.e. herbivores are present. Plants can even integrate environmental cues that predict upcoming herbivory to accelerate or enhance their induced defences which is termed as priming^8,9^. For example volatiles of herbivore-attacked plants can prime herbivory-induced defence in adjacent plants^10^. The egg-deposition of herbivorous insects on a host plant can also prime plant defence against the feeding larvae as has been shown for the wild tobacco *Nicotiana attenuata*^11,12^. Also in tomato, moth oviposition primes the wound-induced expression of defence-related parameters^13^. Priming by insect oviposition is likely more common because several plants possess an increased resistance to insect larvae if previously exposed to its eggs^14–17^. Yet, the larvae may also be affected by defence responses that are directly activated upon insect oviposition. Plant responses to insect oviposition include the formation of neoplasms, necrosis, egg-crushing tissues, ovicidal substances or volatiles that attract egg predators and parasitoids^18–24^.

Several plant responses to oviposition as well as the effects on plant resistance to subsequent larval attack can be specific to the insect species. For example, *Brassica nigra* plants are more resistant to feeding larvae in response to a specialist’s but not a generalist’s moth oviposition^25^, suggesting a differential plant response to oviposition by both insects. Yet, *N. attenuata* plants are more resistant to larvae of only the generalist *Spodoptera exigua* but not the specialist *Manduca sexta* in response to oviposition by either of the two species^12^. Oviposition by both moth species equally primes the feeding-induced plant production of phenylpropanoid-polyamine conjugates (PPCs), which reduces performance of *S. exigua* larvae feeding on oviposited plants^11^. This suggests a differential susceptibility of the larvae but no species-specific plant response to oviposition, though *N. attenuata* may show other, still undiscovered, responses that may differ for the two moth species.

Plant responses to herbivory are multifaceted, which is reflected in a large reprogramming of a plant’s transcriptome and various shifts in its primary and secondary metabolism^26^. This reprogramming is regulated by a network of signalling pathways in which the phytohormone jasmonic acid (JA) plays a central role^27^. Differential activation of these signalling pathways in response to different elicitors, e.g. damage- and herbivore associated molecular patterns, allows species-specific responses to different herbivores^28–30^. For example, larval feeding of *M. sexta* and *S. exigua* elicits distinct transcriptional profiles in *N. attenuata*^4^, likely due to differential phytohormone induction patterns that result from different ratios of elicitors in the larval oral secretions of both species^31^.

The phytohormonal and transcriptional plant responses to insect oviposition are less examined than those to insect feeding but an involvement of salicylic acid (SA) and JA signalling pathways has been inferred^32^. *Arabidopsis thaliana* leaves oviposited by *Pieris brassicae* accumulate high levels of SA^33^ and SA-responsive transcripts^34^. Consistent with the role of SA as a key regulator of plant responses to phytopathogens, the transcriptional profile in response to *P. brassicae* oviposition is more similar to that of plants harbouring a bacterial infection than to plants fed by *P. brassicae* larvae^34^. The SA signal is also detected in systemic leaves of oviposited plants that thereby even acquire resistance (SAR) to bacterial pathogens^35^. JA may also play a role in plant responses to oviposition as indicated by an increased hatching rate of spider mite eggs on JA-deficient tomato mutants^36^. Moreover, JA-biosynthesis genes are induced upon oviposition that involves wounding of plant tissue^37,38^ and plant responses to such oviposition can be mimicked with JA treatments^39,40^.

Very few studies examined the influence of an earlier oviposition on plant signalling in response to the feeding larvae. Transcriptome analyses revealed no significant effects of *P. brassicae* oviposition on *A. thaliana*’s response to larval feeding^41^ or very few *B. nigra* genes that were altered in larval attacked plants due to a pre-treatment with *P. brassicae* egg extract^42^. In tomato, the increase of wound-induced expression of a defensive protease inhibitor (PI) gene in response to a moth’s oviposition coincides with a stronger JA-burst^13^. Yet, in *N. attenuata*, the priming effect of moth oviposition on the induction of PI activity and PPC production in response to larval feeding could not be explained by increased induction of JA^11,43^. Overall, our current knowledge on the signalling of oviposition-induced responses is anecdotal, especially regarding the question how oviposition affects plant responses to subsequently feeding larvae.

**Figure 1:**
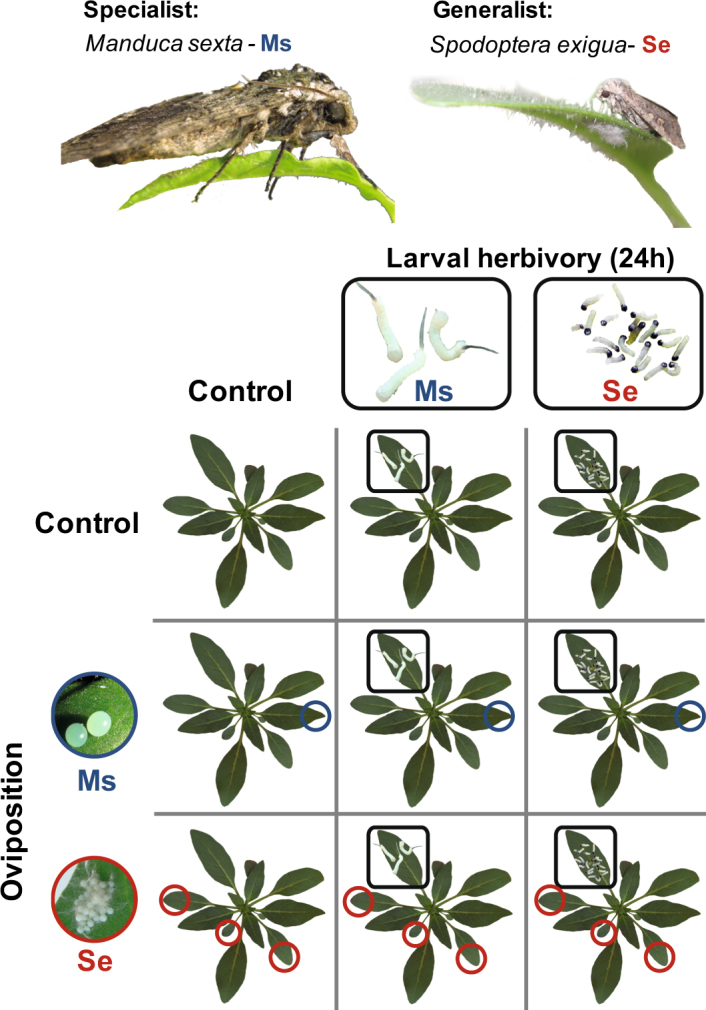
Experimental design. A 3 × 3 full-factorial priming experiment was conducted with *N. attenuata* (12 plants per treatment) to assess the effects of oviposition and larval feeding by the specialist herbivore *M. sexta* (Ms, blue) and the generalist herbivore *S. exigua* (Se, red). Shortly before hatching, the eggs were removed, which was 4 days after oviposition on a standardised leaf position in case of *M. sexta* (blue circles) but after 3 days in case of *S. exigua* that only oviposited on elder leaves (red circles). Neonate larvae were applied at the next day to a standardised leaf position (black square), which was harvested from all plants after the larvae fed for 24 hours.

Here we explore *N. attenuata*’s signalling response to oviposition and herbivory by two of its major herbivores. Specifically, we examine the plant for i) systemic imprints of oviposition by *M. sexta* and *S. exigua*, ii) how the moth’s oviposition affects the feeding-induced plant response to its larvae, and iii) whether these effects of oviposition on plant responses to feeding are specific for the insect species. In a full-factorial design (Fig. 1), we explore the effects of larval feeding and prior oviposition by *S. exigua* and *M. sexta* on *N. attenuata*’s transcriptional and phytohormonal profiles in leaves that were systemic to oviposition but local for the larval attack.

## Results

### Plant responses to oviposition in leaves systemic to the oviposition site

**Figure 2:**
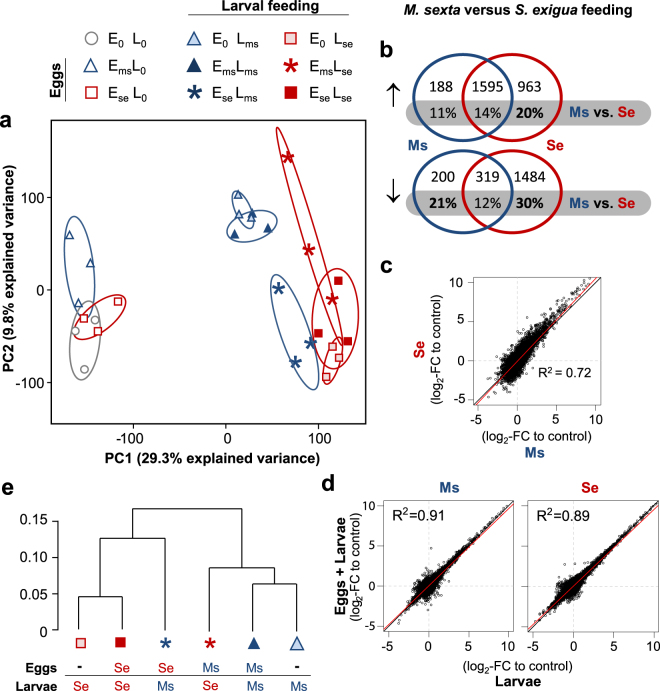
Species-specific effects of oviposition and larval herbivory on *N. attenuata’s* transcriptional regulation. (**a**) Principle component analysis (PCA) of transcript profiles of *N. attenuata* in response to either *S. exigua* (Se) or *M. sexta* (Ms) oviposition and/or larval feeding. The legend above presents all symbols used to depict all treatments in the 3 × 3 factorial design and treatment replicates are outlined by 68% confidence intervals. (**b**) Venn diagram comparing the numbers of genes that were regulated in the microarray analysis by either *S. exigua* or *M. sexta* larval feeding (relative to control plants) and those significantly different between the two (at *P* < 0.05 after correction for false discovery rate and a log_2_-fold change (FC) > 1). (**c**) Scatter plots of the log_2_-FC in gene expression in response to larval feeding by *M. sexta* and *S. exigua*. (**d**) Scatter plots of the log_2_-FC in gene expression in response to larval feeding on plants oviposited and non-oviposited by conspecific moths (left: *M. sexta*, right: *S. exigua*). (**e**) Hierarchical clustering of the log_2_FC in gene expression (relative to control plants) of all feeding-induced treatments.

We evaluated the direct effects of oviposition on plants without larval feeding. These leaves were harvested at the same time and from the same leaf position as the leaves of feeding-induced plants and were therefore systemic to the oviposited leaf (see methods). We found no differences in the phytohormone profiles between oviposited and non-oviposited control plants. Levels of JA and JA-Ile generally varied around the limit of quantification (ca. 7 and 3 ng/g FW, respectively) and are therefore not displayed. Levels of SA and ABA did not differ between plants oviposited by *S. exigua* or *M. sexta* and control plants (see Supplementary Fig. S1). However, the transcriptome profiles of *M. sexta*-oviposited plants (E_ms_L_0_) separated from those of control plants (E_0_L_0_) in a principal component analysis (PCA) along the second principal component (Fig. 2a). In contrast, transcriptome profiles of *S. exigua*-oviposited plants (E_se_L_0_) grouped within those of control plants. Oviposition by *M. sexta*, but not by *S. exigua*, significantly altered the expression of 59 genes, several of which are related to ethylene and auxin signalling as well as regulators of transcription (Supplementary Table S1).

### *N. attenuata* responds species-specific to larval feeding

Altogether, transcriptomes of plants attacked by larvae clearly separated from the non-attacked plants along the first principal component in a PCA (Fig. 2a). Along the second principal component, explaining about 10% of the variation, *S. exigua*-fed plants (E_0_L_se_; filled squares) clearly separated from *M. sexta-*fed plants (E_0_L_ms_; filled triangles).

Herbivory by any of the two species significantly altered the expression of 4749 genes (11% of all genes on the array) relative to control plants (E_0_L_0_). Whereas feeding by *S. exigua* altered the expression of almost 92% (4361) of these genes, *M. sexta* feeding regulated only about half (2302) of them (Fig. 2b). As a consequence, the feeding-induced plant responses to both species overlapped just for 40% (1914). Of all feeding-inducible transcripts, only about 8% (388) were specific to *M. sexta* but almost 52% (2447) were unique for feeding *S. exigua*. In a direct comparison between *S. exigua*-fed (E_0_L_se_) and *M. sexta*-fed (E_0_L_ms_) plants, 20% of the feeding-induced genes differed significantly. The transcriptional changes (log_2_-fold changes; log_2_-FC) relative to untreated controls correlated with an R^2^ of 0.72 between plants fed by larvae of the two different species (Fig. 2c), which is about 20% lower than correlations between plants fed by larvae of the same species (either or not previously oviposited by conspecifics; see below and Fig. 2d).

**Figure 3:**
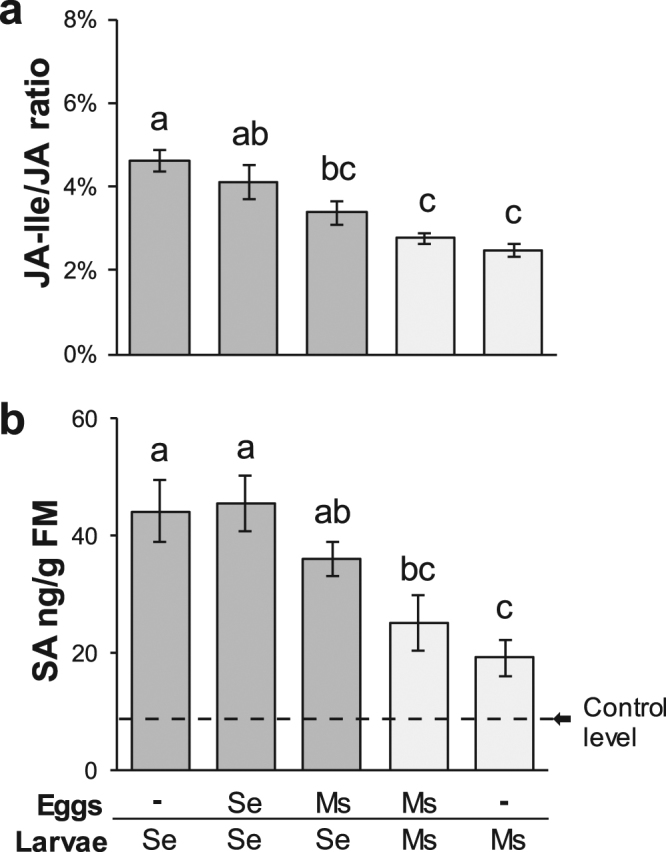
Species-specific effects of larval herbivory on *N. attenuata’s* phytohormone levels. Mean ± SEM (N = 8–10) of (**a**) the ratio of the jasmonic acid (JA) to its conjugate with isoleucine (JA-Ile) and (**b**) salicylic acid (SA) content in *N. attenuata* leaves fed by *S. exigua* (Se) or *M. sexta* (Ms) for 24 hours. The plants were previously either untreated or oviposited at a systemic leaf. An arrow marks the SA level of unattacked control plants. Letters indicate significant differences at *P* < 0.05 according to Kruskal Wallis and pairwise Wilcoxon rank sum test (in case of the JA-Ile/JA ratio) or ANOVA followed by paired *t*-test (in case of SA).

The difference in the transcriptional response to the two herbivore species was accompanied by a different induction of phytohormones. Feeding by both species increased levels of JA, JA-Ile, SA and ABA. In *S. exigua*-fed plants, levels of SA and the ratio of JA-Ile to JA were about twofold higher compared to *M. sexta*-fed plants (see outer bars in Fig. 3), while ABA was altered similarly by both species (Supplementary Fig. S1).

### Effects of oviposition on *N. attenuata’s* response to herbivory by conspecific larvae

Oviposition by neither *M. sexta* nor *S. exigua* altered any of the assessed phytohormone accumulations in response to herbivory by conspecific larvae (Fig. 3, see Supplementary Fig. S1 and Supplementary Table S2). For each of the two species, non-oviposited and previously oviposited plants shared about 75% of the transcriptional regulation in response to feeding by conspecific larvae (relative to control plants; see Supplementary Fig. S2).

To test whether the previous oviposition changed the plant’s transcriptional response to the subsequent feeding by conspecific larvae, we evaluated the effects of previous oviposition and the larval feeding in two-factorial analyses (including E_0_L_0_/E_ms_L_0_/E_0_L_ms_/E_ms_L_ms_ in case of *M. sexta* and E_0_L_0_/E_se_L_0_/E_0_L_se_/E_se_L_se_ plants in case of *S. exigua*), but we found no interactions between oviposition and larval feeding for neither of the two species. Similarly, we found no significant differences in direct comparisons of gene expression in plants only exposed to larval feeding and plants that were oviposited prior to larval herbivory by the same species (E_0_L_ms_ versus E_ms_L_ms_ and E_0_L_se_ versus E_se_L_se_).

In accordance with that, the feeding-induced transcriptional changes (log_2_-FC) relative to control plants correlated very strongly for previously oviposited and non-oviposited plants (Fig. 2d). Also in the PCA, *M. sexta-*fed plants with a prior oviposition by conspecifics (E_ms_L_ms_) cluster closely together with plants only fed by *M. sexta* (E_0_L_ms_) and similarly do *S. exigua-*fed plants with a prior oviposition by conspecifics (E_se_L_se_) and plants only fed by *S. exigua* (E_0_L_se_) cluster together (Fig. 2a).

### Oviposition shapes the species-specific response of *N. attenuata* to larval feeding

We investigated furthermore, whether the plant response to feeding larvae is altered in plants previously oviposited by heterospecific moths. In the PCA, transcript profiles of the E_se_L_ms_ cross-species treatment (blue stars in Fig. 2a) clearly separated from the other two *M. sexta*-fed plant treatments (blue filled triangles) and aggregated with the *S. exigua*-fed treatments (red filled squares). Similarly, transcript profiles of the other E_ms_L_se_ cross-species treatment (red stars) shifted towards plants fed by *M. sexta*. This pattern was further supported by a clustering analysis of the six feeding induced treatments, in which E_se_L_ms_ plants clustered together with *S. exigua-*fed plants that were either oviposited by conspecific moths or not (Fig. 2e). *Vice versa*, E_ms_L_se_ clustered with *M. sexta-*fed plants.

**Figure 4:**
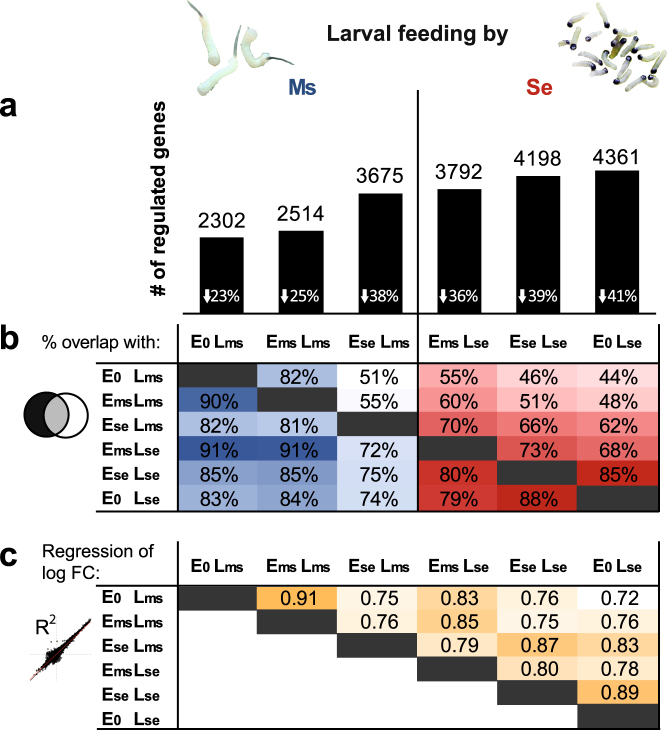
Oviposition by the other species shifted the transcriptional response to feeding by *M. sexta* and *S. exigua* larvae in-between the species-specific plant responses to their herbivory. (**a**) Bars represent the numbers of genes (given above) differentially regulated relative to control plants (FDR-adjusted *P* < 0.05, log_2_-fold change >1) in all feeding-induced plant treatments. The white numbers in the bars display the proportion of down-regulated genes. The plants had experienced egg deposition (E) by *M. sexta* (Ms), *S. exigua* (Se), or none before they were fed by neonate larvae (L) of either of the two species for 24 hours. (**b**) Matrix of the proportions of gene sets similarly regulated by the different feeding-induced treatments: For each treatment designated on top of a column, the proportion of genes that are also regulated by another treatment (designated in front of a row) is given. (**c**) Matrix of the regression coefficients of pairwise correlations between log_2_-FC in gene expression (compared to control plants) among all feeding-induced treatments.

The numbers of genes regulated by the cross-species treatments fall in-between those regulated in plants that only experienced one of the species (Fig. 4a). For example, the number of genes regulated in E_se_L_ms_ plants increased by more than 1000 compared to the other two *M. sexta*-fed plant treatments (E_0_L_ms_, E_ms_L_ms_). As a consequence, E_se_L_ms_ plants shared only 51% of its regulated genes with E_0_L_ms_ plants, while these overlapped with 82% of the genes regulated in E_ms_L_ms_ (Fig. 4b). Instead, genes regulated in E_se_L_ms_ plants overlap with 62% of the genes regulated in E_0_L_se_ plants, which is considerably more than the 44–48% that the other *M. sexta*-fed plants overlapped with the E_0_L_se_ plants.

Similarly, plants of the *S. exigua*-fed cross-species treatment (E_ms_L_se_) had about 500 genes less regulated than the other two *S. exigua*-fed plant treatments. While, E_0_L_se_ and E_se_L_se_ plants overlapped in 85–88% of their regulated genes, E_ms_L_se_ plants overlapped only with 68% of the genes regulated in E_0_L_se_ plants. Conversely, the proportion of *M. sexta*-responsive genes (E_0_L_ms_) that are also altered in response to *S. exigua* feeding increased from 83% (E_0_L_se_) to 91% (E_ms_L_se_) when *M. sexta* oviposition preceded *S. exigua* feeding. Thus, the comparisons of the regulated gene sets support that the plant response in cross-species treatments is intermediate to the species-specific responses to larval feeding.

Finally, the shift in the species-specific response to feeding was also reflected in pairwise regression analyses of the log_2_-FC relative to untreated controls (Fig. 4c, see Supplementary Fig. S3). The strong correlations between E_0_L_se_ and E_se_L_se_ as well as between E_0_L_ms_ and E_ms_L_ms_ with an R^2^ around 0.9 were not found between the cross-species treatments (E_ms_L_se_ and E_se_L_ms_) and the E_0_L_ms_ and E_0_L_se_ treatments. Instead, the expression pattern of E_ms_L_se_ plants correlated similarly with E_0_L_ms_ and E_0_L_se_ (R^2^ of 0.82 and 0.83) and E_se_L_ms_ plants correlated even slightly stronger with E_0_L_se_ than with E_0_L_ms_ plants.

Lastly, the cross-species treatment for which phytohormones were analysed (E_ms_L_se_) showed a phytohormonal induction pattern in-between of E_0_L_ms_ and E_0_L_se_ plants (Fig. 3a,b). The intermediate levels of SA and JA-Ile/JA in E_ms_L_se_ plants did not significantly differ from both treatments with *M. sexta* feeding and JA-Ile/JA levels in E_ms_L_se_ plants even differed significantly from E_0_L_se_ plants.

### Validation of microarray analysis by real-time qPCR of candidate genes

**Figure 5:**
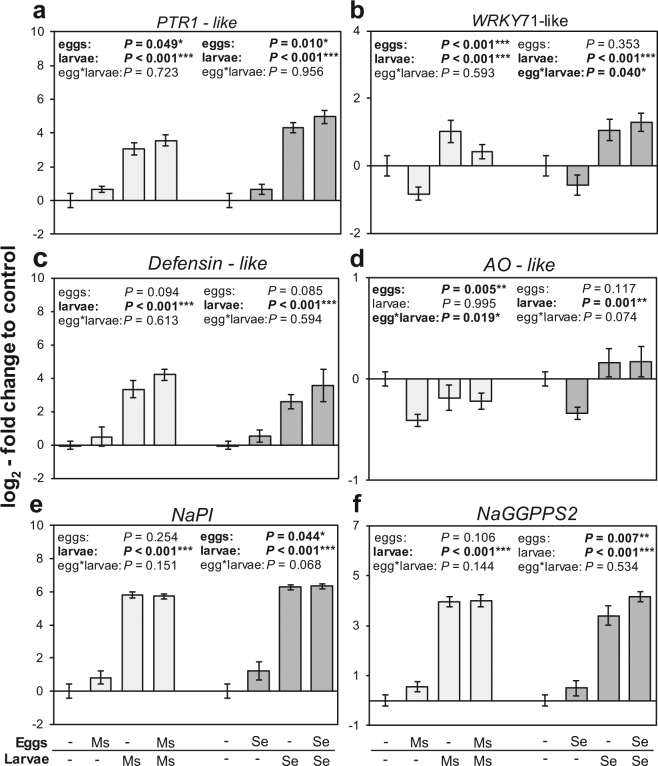
Effects of oviposition and feeding on expression of *N. attenuata* genes. Transcript accumulation (mean ± SEM, N = 9–10) of (**a**) *PTR1* homolog (oligo peptide transporter: Na_454_15271), (**b**) *WRKY* transcription factor (WRKY 71-like: Na_454_21846), (**c**) a defensin-like protein (Na_454_03111), (**d**) L-aspartate oxidase (*AO*: Na_454_00281), (**e**) trypsin protease inhibitor *NaPI* (Na_454_02256) and (**f**) geranylgeranyl diphosphate synthase *NaGGPPS2* (Na_454_01146) in *N. attenuata* plants in response to the oviposition by and later the feeding larvae of either *S. exigua* or *M. sexta*. Transcript accumulation relative to untreated plants was determined by real-time qPCR normalized to *β-actin* and *EF1α* as reference genes. Asterisks indicate significant differences at *P* < 0.05/0.005/0.001 according to linear mixed-effects models with oviposition and larvae of either *S. exigua* or *M. sexta* as fixed factors and replicate blocks as random factor.

**Figure 6:**
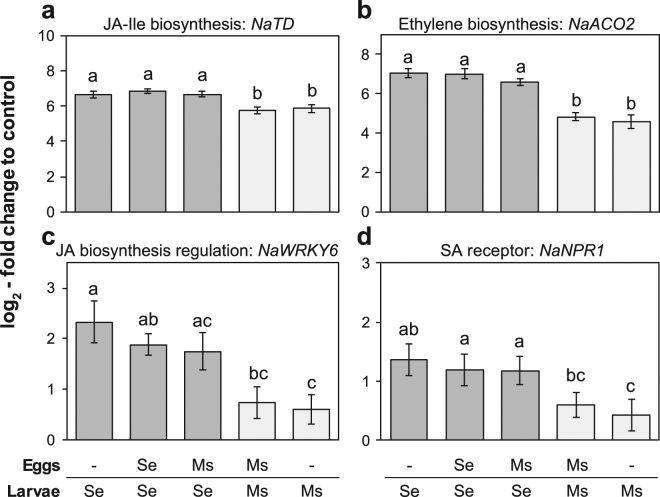
*N. attenuata* signalling genes differentially induced by larvae of *M. sexta* and *S. exigua*. Transcript accumulation (mean ± SEM) of selected genes known to be key regulators in different plant signalling pathways: (**a**) *threonine deaminase (NaTD:* Na_454_03285), (**b**) *aminocyclo-propanecarboxylate oxidase* (*NaACO2:* Na_454_06634), (**c**) transcription factor *WRKY*6 (Na_454_14052), (**d**) SA receptor (*NaNPR1:* Na_454_12995) in plants fed by either *S. exigua* (Se, dark bars) or *M. sexta* (Ms, light bars) larvae. The plants were previously oviposited by either of the two species or not (−). Transcript accumulation relative to untreated plants was determined by real-time qPCR and normalized to *β-actin* and *EF1α* as reference genes. Letters indicate significant differences at *P* < 0.05 according to linear mixed-effects models with the different herbivory treatments as fixed factors and replicate blocks as random factor followed by a Tukey post-hoc test (N = 9–10).

**Figure 7:**
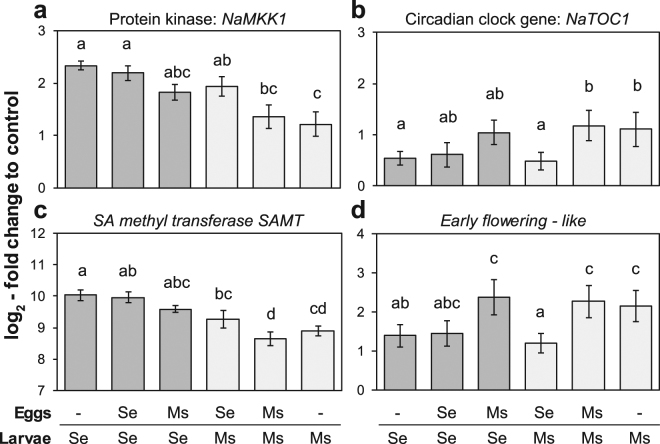
*N. attenuata* genes shifted by oviposition in their species-specific feeding-induced expression. In plants fed by either *S. exigua* (Se, dark bars) or *M. sexta* (Ms, light bars) larvae, transcript accumulation (mean ± SEM) of (**a**) the *mitogen-activated protein kinase kinase (NaMKK1:* Na_454_10210), (**b**) a *SA methyl transferase (SAMT:* Na_454_08320), (**c**) the circadian clock gene *timing of CAB expression (NaTOC1:* Na_454_09895), and (**d**) an *early flowering-like* gene (Na_454_09170) was determined by real-time qPCR and normalized to *EF1α* as reference gene and expressed relative to untreated control plants. Plants were previously oviposited by either of the two species or not (−) and letters indicate significant differences at *P* < 0.05 according to linear mixed-effects models with the different herbivory treatments as fixed factors and replicate block as random factor followed by a Tukey post-hoc test (N = 9–10).

We quantified transcripts in 9-10 biological replicates for a set of genes that we selected based on their regulation in the microarray analysis or that are known to be key regulators in different plant signalling pathways. We selected (i) genes that were significantly regulated by the oviposition alone (Supplementary Table S3) or that indicated an altered regulation after larval feeding due to prior oviposition by the same species (Fig. 5), (ii) signalling-related genes that may reflect the species-specific response to the larval feeding of both species (Fig. 6), and (iii) genes that signify the shift of the species-specific feeding-induced response by the other species’ oviposition (Fig. 7). Overall, we validated the expression patterns determined by the microarray analysis for all 17 genes by real-time qPCR (Supplementary Figs S4 and S5).

Our analyses verified the up-regulation of the oligo peptide transporter *PTR1*-like in response to the oviposition by both species whereas up-regulation of a *defensin*-like protein by *M. sexta* oviposition was only indicated by trend (Fig. 5). Plants with *M. sexta* oviposition had significantly reduced transcript levels of a predicted *WRKY71* transcription factor and an L-aspartate oxidase gene (*AO*). While plants with *S. exigua* oviposition had increased transcript levels of the trypsin protease inhibitor gene *NaPI* and the geranylgeranyl diphosphate synthase gene *NaGGPPS2*.

Transcripts of *PTR1* and *defensin* also increased in response to larval feeding by both species, which tended to be stronger when the plants were previously oviposited by conspecifics. Though not statistically significant, this could point towards additive effects of oviposition and feeding. The increased transcript accumulation of the *NaPI* gene in response to *S. exigua* oviposition did also not result in increased *NaPI* transcripts after larval feeding compared to only feeding-damaged plants (Fig. 5e). In contrast, dual comparisons between feeding-induced plants revealed significantly increased transcripts of *NaGGPPS2* in *S. exigua*-oviposited compared to only feeding-induced plants (Fig. 5f). Transcript levels of the *WRKY71*-like transcription factor and the *AO* gene were similar in feeding-induced plants whether the plants were oviposited or not. But since these genes were down-regulated in only-oviposited plants, they show a significant interaction between oviposition and larval feeding by *S. exigua* and *M. sexta* respectively (Fig. 5b,d). In summary, 24 hours after onset of larval feeding, we could still determine some genes affected by the previous oviposition *per se* but we could not pin down the effect of prior oviposition by conspecific moths on feeding-induced gene expression.

We additionally analysed a set of well characterised *N. attenuata* genes that play a role in different plant signalling pathways related to herbivory. These genes are involved in the regulation of the JA pathway (*NaLOX3*, *NaTD*, *NaWRKY6*), biosynthesis of ethylene (*NaACO2*) and of SA signalling (*NaNPR1*). All of these genes were significantly induced by larval feeding but not by oviposition and oviposition did not affect the level of induction after feeding (Fig. 6). They all showed a higher transcript accumulation in response to *S. exigua* than to *M. sexta*, except for *NaLOX3*, which was not differently regulated between the two species (Supplementary Fig. S4).

As oviposition shifted the species-specific transcriptomic imprint of the larval feeding, we also verified such a transcriptional shift for a selected set of genes. Three of them, the *mitogen-activated protein kinase kinase NaMKK1*, the *SA methyl transferase SAMT* and the *1-aminocyclopropane-1-carboxylic acid oxidase NaACO2* were stronger upregulated in response to *S. exigua* feeding than in response to *M. sexta* feeding but showed an intermediate expression to feeding by both larvae when they were previously oviposited by the other species (Fig. 7, Supplementary Fig. S5, Supplementary Table S4). Three other genes, the circadian clock gene *timing of CAB expression NaTOC1*, an *early flowering-like* gene and a gene homologous to *Jumonji C-domain-containing proteins, JmjC30-like* showed a higher expression to *M. sexta* than to *S. exigua* feeding, which was switched when the plant was previously oviposited by the other species.

## Discussion

We examined whether the oviposition *per se* left imprints in the plant’s phytohomonal and transcriptional regulation in a systemic leaf. In line with previous studies^11,12^, we found no differences in phytohormone levels between oviposited and control plants. The transcript profile in plants oviposited by *S. exigua* did also not differ from that of control plants, but our microarray analysis revealed an imprint of *M. sexta* oviposition on *N. attenuata*’s transcriptome. Even a day after the end of the egg incubation time, expression of some genes was significantly altered and many of them were annotated to signalling and regulatory functions (e.g. transcription factors, hormone biosynthesis, see Supplementary Table S1). Moreover, about half of these genes were also altered in response to *M. sexta* feeding (see Supplementary Fig. S2). This is consistent with the idea that also subtle changes in gene regulation can be biologically relevant^44^.

We identified several genes responsive to oviposition by both *M. sexta* and *S. exigua* in our better replicated qPCR analysis (Fig. 5). Among them are genes involved in plant defence, for example *NaGGPPS* and *NaPI*^45,46^. These were induced by *S. exigua*- but not by *M. sexta*-oviposition. Interestingly, PI activity in response to larval feeding was also increased in *S. exigua*- but not in *M. sexta*-oviposited plants^11,12^. However, compared to the effect of larval feeding, oviposition only slightly induced *NaPI* expression and whether that could explain the priming of feeding-induced PI activity remains an open question.

Generally, it is not surprising that one day after egg-removal, systemic *N. attenuata* leaves did not display the large extent of changes in gene expression as described for the local leaves of *A. thaliana* 24–72 hours after oviposition by *Pieris* moths^34^. Even in local *A. thaliana* leaves, no transcriptional imprint was detected anymore, one day after egg-removal^41^. Yet, both plant species differ in their physiological response to insect oviposition. Other than *N. attenuata*, *A. thaliana* responds to oviposition with HR-like symptoms^34^ which can result in SAR against phytopathogenic bacteria^33,35^. Whereas oviposition by the specialist *P. brassicae* induces HR-like necrosis in many brassicaceous plants^17,21^, oviposition by the generalist *Mamestra brassicae* does not, nor does the latter affect larval performance^25^. However, in *N. attenuata*, oviposition by both a specialist and a generalist moth species prime feeding-induced defences and lower the performance of *S. exigua* larvae^11,12^. In line with that, all defence-related candidate genes that were affected by oviposition in our qPCR analysis showed similar tendencies for the oviposition by both moth species even if the effect was only significant for one of the two species.

As expected, *N. attenuata* exhibited diverging responses to the feeding larvae of both species. Larvae of *S. exigua* induced more SA than those of *M. sexta*, just as had been previously described^31^. Contrasting this earlier study^31^, *M. sexta* feeding also induced some SA and *S. exigua*-fed plants showed no lower JA induction but a greater JA-Ile/JA ratio than *M. sexta*-fed plants, which may even suggest a higher conversion of JA to the biologically active conjugate. However, in line with our results, a recent study^47^ also describes SA induction after *M. sexta* feeding and an equal JA-elicitation for *N. attenuata*’s response to *M. sexta* and *Spodoptera littoralis*. Consistent with our phytohormone analysis, transcripts of the Ile-biosynthesis gene *NaTD* as well as the SA-receptor *NaNPR1* were differentially activated by larval feeding of both species, whereas that of the JA-biosynthesis gene *NaLOX3* was not (Fig. 6, Supplementary Fig. S4). Thus, our results support that SA signalling plays a role in tuning *N. attenuata*’s species-specific response to larval feeding, but this tuning is not necessarily linked to JA-SA antagonism as had been suggested earlier^31^.

The distinguished species-specific response to the feeding larvae was reflected in the number of regulated genes as well as in PCA, regression and cluster analyses of the plant’s transcriptome (Figs 2–4). Our results match well with those of a study that pioneered *N. attenuata*’s species-specific transcriptional response to different herbivores^4^ using cDNA microarrays displaying only 240 genes with a bias for herbivory-related genes. Like this earlier study^4^, our unbiased transcriptome microarray, showed that a large fraction of feeding-responsive genes was specifically regulated by feeding of the generalist *S. exigua* and this fraction was greater among down- than among up-regulated genes. In line with this, the shared transcriptional response to two generalist herbivores *Heliothis virescens* and *S. exigua* differentiating from the response to *M. sexta* is also dominated by down-regulated genes^4^. Differential plant responses to herbivores of the same feeding guild but different diet breadth are also apparent in other plant-insect interactions, which could be either adaptive for the plant or the result of host manipulation by insects^5–7,26^. Presumably, generalist herbivores are more likely to evolve mechanisms to suppress plant defences than specialists as they have to cope with diverse plant defences^7^. However, *S. exigua* feeding altered more than 2000 genes more than *M. sexta* feeding. Although, this was more pronounced for down-regulated genes, defence-related genes and signalling pathways were often stronger induced in response to the generalist (Figs 2–7) and the majority of genes down-regulated after herbivory are usually related to primary plant metabolism^48^. *N. attenuata* effectively defends itself against *S. exigua*^11,46,49^, whereas *M. sexta* is relatively tolerant to many of its direct defences^47,50^. Thus, it is more likely that the differential plant response is driven either by the specialist herbivore or the plant itself.

The distinct response of *N. attenuata* to both herbivores is likely due to the divergent composition of elicitors in their oral secretions^31^. The receptors recognizing these elicitors have not been identified so far but they presumably activate mitogen-activated protein (MAP) kinases, usually three in a cascade, that mediate the transduction of the signal. We found that one of the MAP kinases (*NaMKK1*) that are involved in the regulation of *N. attenuata*’s defence response to herbivory^51^, reflected the species-specific expression pattern (Fig. 7). Even though *NaMKK1* is not required for feeding-induced JA and ethylene production, it fine-tunes *N. attenuata*’s defence response^52^ and thus it may also be involved in adjusting the plant’s response to the attacking herbivore species.

To examine plant responses that may be associated with *N. attenuata*’s oviposition-primed defence response to larval feeding^11^, we compared only feeding-induced plants with plants that had been additionally oviposited by conspecific moths. Yet, we could not determine how insect oviposition on *N. attenuata* alters feeding-induced plant defence metabolites and the performance or immune state of *S. exigua* and *M. sexta* larvae, respectively^11,12^. The transcriptional responses between only feeding-induced plants and plants with prior oviposition by conspecifics correlated to 90%, which only emphasizes the high reproducibility of the herbivory treatments within our microarray analysis. However, despite their biological relevance, subtle changes in only few genes can be easily overlooked due to the large number of variables that are analysed with a very limited number of replicates^44^. For example, pre-treatments with phytopathogenic bacteria or egg extract that both have a negative impact on larval performance of *P. brassicae*, show just very subtle effects on the transcriptional response of *B. nigra* to larval feeding^42^.

That we could not determine signalling components which could explain these effects may be due to one or a combination of the following reasons: The regulation of the oviposition-priming of plant defence may (i) occur at different levels, e.g. post-transcriptionally, (ii) involve only few key components at an effect-size that is too small to be revealed in a low-replicated multi-variate approach, and (iii) occur at other time points after the onset of larval feeding. Priming of feeding-induced responses may result in an earlier, faster, stronger, more sensitive or even qualitatively different response on the molecular level^9^. In order to minimize variation between plant-replicates, that may be driven by random differences in the feeding behaviour of the larvae on a shorter time scale, we examined a time point at which the feeding-induced response on the transcriptional and phytohormonal level is well settled. However, thereby we may have missed the time point at which the plant defence priming by oviposition is regulated, for instance if it would cause an earlier or faster plant response just upon the larvae started feeding.

Opposite to prior oviposition by conspecifics, our results revealed that oviposition by one species altered the species-specific feeding response to larvae of the other species. In both cross-species treatments, the plant’s response to feeding larvae became intermediate in those characteristics that were specific for the larval feeding of both species. This was consistently apparent in all analyses of *N. attenuata*’s transcriptional response (PCA, cluster and regression analyses as well as the numbers and overlaps of significantly regulated genes) as well as in the phytohormone data for SA and JA-Ile/JA (Figs 2–4).

The oviposition-dependent shift of the species-specific gene expression in response to larval feeding was further confirmed by qPCR on six selected genes. Half of them were higher expressed in response to *S. exigua* than to *M. sexta* and showed an intermediate expression in the cross-species treatments (Fig. 7, Supplementary Fig. S5). These genes (*ACO*, *SAMT*, *NaMKK1*) are related to signalling pathways that mediate plant defence. Again, as *NaMKK1* is fine-tuning *N. attenuata*’s defence response^52^, it may also play a role in the oviposition-mediated shift of the plant response to larval feeding. Three circadian clock genes were stronger expressed after *M. sexta*- than after *S. exigua*-feeding but showed the reversed pattern in the cross-species treatments. Two of them were also induced by *M. sexta* oviposition at levels comparable to feeding (Supplementary Table S1). Previous studies suggested that diurnal regulation plays a role in orchestrating plant anti-herbivore defences^53,54^ and this may be the case for the oviposition-mediated responses we found in *N. attenuata* as well.

Attack by multiple herbivores modifies plant responses in a non-additive manner and consequently can strongly affect the outcome of plant-insect interactions^55^. Our study shows that such interactions between different insects can begin before actual herbivory occurs. That insect oviposition alters plant resistance to the ovipositing species is well-established today^56^ while the notion that also other biotic stressors can be affected is just emerging^12,35^.

Our study revealed that insect oviposition can shape the species-specificity of a plant’s response to feeding larvae. What are possible explanations of this surprising phenomenon? On the one hand, *N. attenuata* may prepare its feeding-induced response to defend most effectively against the herbivore that announced itself by its oviposition. On the other hand, herbivores may start to manipulate their host plant right upon oviposition. The latter scenario may be more likely for the specialist, yet the shift of the species-specific plant response was more pronounced in response to the generalist’s oviposition. Despite its wide host plant range, *S. exigua* is a frequent herbivore in the native habitat of *N. attenuata*^49^ and as such it may exert considerable selection pressure on the plant. If the plant would benefit from rendering its response specific to the insect species, the effect that oviposition can determine this species-specificity connotes a novel mechanism of defence priming.

## Methods

### Plants and insects

Seeds of a *Nicotiana attenuata* Torr. ex Watson inbred line (15x) from a field collection were sterilized and smoke-germinated on agar plates and seedlings were transferred to potting soil and grown in 1.5 L pots in a greenhouse (24 °C (±10):15 °C; 16:8 h L:D) as described earlier^11^. *Manduca sexta* L. and *Spodoptera exigua* Hübner were cultured in a climate chamber (24 °C; 16:8 L:D). Insect cultures and the wheat germ based artificial diets of the larvae were described earlier^57^.

### Experimental procedure

We assigned 5-week-old early elongated experimental plants of equal size and elongation state to one of nine treatments for each of 12 biological replicate blocks (Fig. 1). In a 3 × 3 full-factorial experimental design, we examined the plant responses to oviposition and larval feeding by *M. sexta* and *S. exigua* and the interactive effects between them. The setup of the oviposition and larval feeding treatments were previously described in detail^58^. In brief, we exposed the second youngest source leaf (2 positions below the sink-source transition leaf) through slots in the side of a flight cage to *M. sexta* oviposition. As *S. exigua* does not accept this setup for oviposition, we exposed whole plants to oviposition by *S. exigua* for 12 h. *M. sexta*-oviposited plants received 2.2 ± 0.4 (mean ± SEM) and *S. exigua*-oviposited 37.7 ± 5.6 eggs (dispersed on one to four elder leaves). Within the range of the natural egg incubation time, 3 and 4 days after oviposition by *S. exigua* and *M. sexta* respectively, we removed the eggs of all plants without damaging the leaf surface either with a soft brush or a featherweight forceps.

Within 12 h after egg-removal, we applied either three *M. sexta* or 20 *S. exigua* neonates per plant to a standardised leaf position relative to the sink-source transition leaf. It was the next younger leaf above the *M. sexta*-oviposited leaf and the corresponding leaf position for control or *S. exigua*-oviposited plants. Due to the size difference of both species, these numbers of larvae inflict comparable amounts of damage. Larvae were kept on the leaf in vented clip cages and plants without herbivory treatments received empty clip cages at corresponding leaf positions. After 24 hours of larval feeding, the standardised leaf (either exposed to larvae or empty clip cages) was harvested from all plants, flash-frozen in liquid nitrogen and stored at −80 °C. Aliquots of the leaf material powdered using mortar and pestle under liquid nitrogen were weighed into separate tubes.

### Extraction and analysis of phytohormones

As previously described in more detail^59^, we extracted about 150 mg of the samples in 1 mL ethyl acetate spiked with deuterated internal standards of JA, JA-Ile, SA, ABA and re-eluted the dried extracts in 400 µL 70% methanol with 0.1% formic acid before analysis by UPLC-ESI-MS/MS (Synapt G2-S HDMS; Waters®, Milford, Massachusetts, USA). After separation on a C18-column (Acquinity UPLC BEH-C18) with water and methanol (both containing 0.1% formic acid) as eluents in gradient mode (250 μL min^−1^), phytohormones were quantified (negative ionization mode, parent/daughter ion selections for JA: 209/59, JA-Ile: 322/130, SA: 137/93, ABA: 263/153, D6-JA: 215/59, D6-JA-Ile: 328/130, D4-SA: 141/97, D6-ABA: 269/159) according to the peak areas of the respective fragment ions relative to that of the internal standards using MassLynx^TM^ (4.1, Waters).

### RNA extraction

We extracted total RNA from 100 mg leaf powder of all individual plants (including those of the E_se_L_ms_ treatment) with TRIzol® Reagent before the precipitated RNA (using 1.2 M sodium chloride and 0.8 M sodium citrate) was DNase digested using TURBO DNA-free™ (both Ambion^TM^, Thermo Fisher Scientific, Waltham, MA USA; http://www.thermofisher.com) according to the manufacturer’s instructions. After a clean-up of the RNA using NucleoSpin® RNA (Macherey-Nagel, Düren, Germany; http://www.mn-net.com/), we adjusted the RNA to 200 ng/µL based on photometric concentration measurements (μDrop™ Plate; Multiskan™ GO Microplate Spectrophotometer, Thermo Scientific).

### Microarray analysis

RNA of 3-4 plant replicates per treatment was pooled at equal proportions resulting in 3 biological replicates for each treatment on the microarray (performed by Oaklabs, http://www.oak-labs.com/). All samples had a RNA integrity index between 7.7 and 8.0 as determined with a 2100 Bioanalyzer (Agilent Technologies, Santa Clara, California, USA, http://www.agilent.com). Fluorescent cRNA was generated using the Low Input QuickAmp Labeling Kit (Agilent Technologies) and oligo-dT primers following the manufacturer’s protocol. Of the cyanine 3-CTP-labelled cRNA, 600 ng were hybridised using the Agilent Gene Expression Hybridisation Kit (Agilent Technologies) following the manufacturer’s protocol on the microarray at 65 °C for 17 h. After the microarray was washed twice, the fluorescence signals on microarrays were detected by the SureScan Microarray Scanner (Agilent Technologies) at a resolution of 3 micron per pixel. The 8 × 60 K microarray harboured the same 60-mer oligonucleotide probes as the Agilent 4 × 44 K microarray with the GEO accession no. GPL13527^60^ that was designed based on the *N. attenuata* transcriptome (BioProject PRJNA223344 at http://www.ncbi.nlm.nih.gov/). Although all RNA samples were processed at once, a first hybridisation on three arrays (24 samples) included only one cross-species treatment. The E_ms_L_se_ was chosen because oviposition by *M. sexta* affects larval performance of *S. exigua* but not *vice versa*^12^. The analysis revealed that, opposite to the effect of prior oviposition of the same species that later fed on the plants, prior exposure to *M. sexta* oviposition shifted the response to larval feeding by *S. exigua*. Therefore, a second analysis including the E_se_L_ms_ treatment was performed.

### Real-time qPCR analysis

To verify microarray results and to test the effects of oviposition and larval feeding on potentially regulated genes with a higher statistical power, we performed real-time qPCR on 9-10 individual plants of each treatment. The verification of the qPCR data was initially performed for 11 genes on the same 8 treatments used in the first microarray hybridisations and was then extended for 6 genes to all feeding-induced treatments including both cross-species treatments and control plants. We synthesized cDNA from the total RNA with the Reverse Transcriptase Core kit (Eurogentec, Seraing, Belgium, http://www.eurogentec.com) according to the manufacturer’s instructions. For each gene, 1 µL cDNA was used in 10 µL reactions of SYBR®Green I-based real-time PCR using a qPCR kit without ROX (Eurogentec) on a StratageneTM Mx3005P® instrument (Agilent Technologies). Real-time PCR was performed with gene-specific primers (see Supplementary Table S5) in triplicates with all treatments of a replicate on one 96 well plate. We inspected melting curves and used LinRegPCR (http://LinRegPCR.HFRC.nl) to determine amplification efficiencies and the relative quantities (RQ) of each gene^61^. The RQ of the genes of interest was normalised to the averaged RQ of two reference genes *β-actin* and *EF1α* (NRQ). Transcript accumulations were analysed on the log_2_-normalized NRQs and depicted relative to the mean of the control treatment.

### Data analysis

All statistics were performed in R version 3.2.3 with the packages *lme4, multcomp*, *psych* and *stats* (http://www.r-project.org). Phytohormone and qPCR data were checked for normality and variance homogeneity (*QQ*-plots and Bartlett’s test) before their statistical evaluation. To test for differences in phytohormone levels between oviposited and control plants and for differences among the five groups of plants exposed to larval feeding, we used either ANOVA followed by paired *t*-tests or Kruskal Wallis test followed by pairwise Wilcoxon rank sum tests. To evaluate the effect of the factors oviposition and larval feeding as well as their interaction within the 2 × 2 full-factorial priming experiments for each species separately we used linear mixed-effects models (LMMs) and included the replicate blocks as random factor. We also analysed transcript accumulations determined by qPCR with LMMs to evaluate differences between control and oviposited plants as well as those between plants of the different larval feeding treatments.

We used the *limma* package^62^ to analyse the microarray data. To reduce background noise, a detection limit was set at the 10% quantile of fluorescence signal intensity of each array. All structural spots, as well as oligos that were not above this detection limit in any of the arrays, were removed from further analysis. Subsequently, the expression levels were background-corrected using the *normexp* method^63^ and normalized between the arrays using quantile normalisation^64^. Principal component analysis was performed on normalised fluorescence signals using *prcomp* and visualised using *ggplot2*^65^. We performed hierarchical clustering using *hcust* and analysed gene expression using *lmFit*. Genes were assumed to be significantly regulated when they differed between treatments by a log_2_-fold change of at least 1 and at *P*-values of below 0.05 after correction for false discovery rate according to Benjamini-Hochberg. Genes of interest were functionally annotated according to NCBI nucleotide blast and blastx of the corresponding p454 sequences (BioProject PRJNA223344).

### Accession codes

The microarray data are available at NCBI Gene Expression Omnibus under accession GSE116273.

## Electronic supplementary material

Supplementary Information

## References

[1] Schaller, A. *Induced plant resistance to herbivory* (Springer, 2008).

[2] Karban R, Agrawal AA (2002). Herbivore offense. Annu. Rev. Ecol. Syst..

[3] Agrawal AA, Petschenka G, Bingham RA, Weber MG, Rasmann S (2012). Toxic cardenolides: Chemical ecology and coevolution of specialized plant-herbivore interactions. New Phytol..

[4] Voelckel C, Baldwin IT (2004). Generalist and specialist lepidopteran larvae elicit different transcriptional responses in *Nicotiana attenuata*, which correlate with larval FAC profiles. Ecol. Lett..

[5] Vogel Hea (2007). Different transcript patterns in response to specialist and generalist herbivores in the wild *Arabidopsis* relative *Boechera divaricarpa*. PLoS. ONE.

[6] Zong N, Wang C-Z (2007). Larval feeding induced defensive responses in tobacco: comparison of two sibling species of *Helicoverpa* with different diet breadths. Planta.

[7] Ali JG, Agrawal AA (2012). Specialist versus generalist insect herbivores and plant defense. Trends Plant Sci..

[8] Conrath U, Beckers GJM, Langenbach CJG, Jaskiewicz MR (2015). Priming for enhanced defense. Annu. Rev. Phytopath..

[9] Hilker M (2016). Priming and memory of stress responses in organisms lacking a nervous system. Biol. Rev..

[10] Heil M, Kost C (2006). Priming of indirect defences. Ecol. Lett..

[11] Bandoly M, Hilker M, Steppuhn A (2015). Oviposition by *Spodoptera exigua* on *Nicotiana attenuata* primes induced plant defense against larval herbivory. Plant J..

[12] Bandoly M, Grichnik R, Hilker M, Steppuhn A (2016). Priming of anti-herbivore defence in *Nicotiana attenuata* by insect oviposition: Herbivore specific effects. Plant Cell Environ..

[13] Kim J, Tooker JF, Luthe DS, De Moraes CM, Felton GW (2012). Insect eggs can enhance wound response in plants: A study system of tomato *Solanum lycopersicum* L. and *Helicoverpa zea* Boddie. PLoS. ONE.

[14] Austel N, Eilers EJ, Meiners T, Hilker M (2016). Elm leaves “warned” by insect egg deposition reduce survival of hatching larvae by a shift in their quantitative leaf metabolite pattern. Plant Cell Environ..

[15] Beyaert I (2012). Can insect egg deposition ‘warn’ a plant of future feeding damage by herbivorous larvae?. Proc. R. Soc. B.

[16] Geiselhardt S (2013). Egg laying of cabbage white butterfly (*Pieris brassicae*) on *Arabidopsis thaliana* affects subsequent performance of the larvae. PLoS. ONE.

[17] Pashalidou FG, Fatouros NE, van Loon JJA, Dicke M, Gols R (2015). Plant-mediated effects of butterfly egg deposition on subsequent caterpillar and pupal development, across different species of wild Brassicaceae. Ecol. Entomol..

[18] Bittner N, Trauer-Kizilelma U, Hilker M (2017). Early plant defence against insect attack: involvement of reactive oxygen species in plant responses to insect egg deposition. Planta.

[19] Desurmont GA, Weston PA (2011). Aggregative oviposition of a phytophagous beetle overcomes egg-crushing plant defences. Ecol. Entomol..

[20] Doss RP (2000). Bruchins: Insect-derived plant regulators that stimulate neoplasm formation. Proc. Natl. Acad. Sci. USA.

[21] Fatouros, N. E. *et al*. Synergistic effects of direct and indirect defences on herbivore egg survival in a wild crucifer. *Proc. R. Soc. B* **281**, 10.1098/rspb.2014.1254 (2014). 10.1098/rspb.2014.1254 PMC4100524 25009068

[22] Geuss D, Stelzer S, Lortzing T, Steppuhn A (2017). *Solanum dulcamara*’s response to eggs of an insect herbivore comprises ovicidal hydrogen peroxide production. Plant Cell Environ..

[23] Petzold-Maxwell J, Wong S, Arellano C, Gould F (2011). Host plant direct defence against eggs of its specialist herbivore. Heliothis subflexa. Ecol. Entomol..

[24] Seino Y, Suzuki Y, Sogawa K (1996). An ovicidal substance produced by rice plants in response to oviposition by the whitebacked planthopper, *Sogatella furcifera* (HORVATH) (Homoptera: Delphacidae). Appl. Entomol. Zool..

[25] Pashalidou FG, Lucas-Barbosa D, van Loon JJA, Dicke M, Fatouros NE (2013). Phenotypic plasticity of plant response to herbivore eggs: effects on resistance to caterpillars and plant development. Ecology.

[26] Appel HM (2014). Transcriptional responses of *Arabidopsis thaliana* to chewing and sucking insect herbivores. Frontiers in Plant Science.

[27] Pieterse CMJ, Van der Does D, Zamioudis C, Leon-Reyes A, Van Wees SCM (2012). Hormonal modulation of plant immunity. Annu. Rev. Cell Develop. Biol..

[28] Bonaventure G (2012). Perception of insect feeding by plants. Plant Biol..

[29] Heil M (2009). Damaged-self recognition in plant herbivore defence. Trends Plant Sci..

[30] Schmelz EA, Engelberth J, Alborn HT, Tumlinson JH, Teal PEA (2009). Phytohormone-based activity mapping of insect herbivore-produced elicitors. Proc. Natl. Acad. Sci. USA.

[31] Diezel C, von Dahl CC, Gaquerel E, Baldwin IT (2009). Different lepidopteran elicitors account for cross-talk in herbivory-induced phytohormone signaling. Plant Physiol..

[32] Reymond P (2013). Perception, signaling and molecular basis of oviposition-mediated plant responses. Planta.

[33] Bruessow F, Gouhier-Darimont C, Buchala A, Metraux JP, Reymond P (2010). Insect eggs suppress plant defence against chewing herbivores. Plant J..

[34] Little D, Gouhier-Darimont C, Bruessow F, Reymond P (2007). Oviposition by pierid butterflies triggers defense responses in. Arabidopsis. Plant Physiol..

[35] Hilfiker O (2014). Insect eggs induce a systemic acquired resistance in *Arabidopsis*. Plant J..

[36] Ament K, Kant MR, Sabelis MW, Haring MA, Schuurink RC (2004). Jasmonic acid is a key regulator of spider mite-induced volatile terpenoid and methyl salicylate emission in tomato. Plant Physiol..

[37] Buchel, K. *et al*. An elm EST database for identifying leaf beetle egg-induced defense genes. *BMC Genomics* **13**, 10.1186/1471-2164-13-242 (2012). 10.1186/1471-2164-13-242 PMC3439254 22702658

[38] De Puysseleyr V, Hofte M, De Clercq P (2011). Ovipositing *Orius laevigatus* increase tomato resistance against *Frankliniella occidentalis* feeding by inducing the wound response. Arthropod Plant Interact..

[39] Hilker M, Kobs C, Varama M, Schrank K (2002). Insect egg deposition induces *Pinus sylvestris* to attract egg parasitoids. J. Exp. Biol..

[40] Meiners T, Westerhaus C, Hilker M (2000). Specificity of chemical cues used by a specialist egg parasitoid during host location. Entomol. Exp. Appl..

[41] Firtzlaff V, Oberländer J, Geiselhardt S, Hilker M, Kunze R (2016). Pre-exposure of *Arabidopsis* to the abiotic or biotic environmental stimuli ‘chilling’ or ‘insect eggs’ exhibits different transcriptomic responses to herbivory. Sci. Rep..

[42] Bonnet C (2017). Combined biotic stresses trigger similar transcriptomic responses but contrasting resistance against a chewing herbivore in *Brassica nigra*. BMC Plant Biol..

[43] Kaur H, Heinzel N, Schottner M, Baldwin IT, Galis I (2010). R2R3-NaMYB8 regulates the accumulation of phenylpropanoid-polyamine conjugates, which are essential for local and systemic defense against insect herbivores in *Nicotiana attenuata*. Plant Physiol..

[44] Sanchez DH, Szymanski J, Erban A, Udvardi MK, Kopka J (2010). Mining for robust transcriptional and metabolic responses to long-term salt stress: a case study on the model legume *Lotus japonicus*. Plant Cell Environ..

[45] Jassbi AR, Gase K, Hettenhausen C, Schmidt A, Baldwin IT (2008). Silencing geranylgeranyl diphosphate synthase in *Nicotiana attenuata* dramatically impairs resistance to tobacco hornworm. Plant Physiol..

[46] Steppuhn A, Baldwin IT (2007). Resistance management in a native plant: nicotine prevents herbivores from compensating for plant protease inhibitors. Ecol. Lett..

[47] Xu S, Zhou W, Pottinger S, Baldwin IT (2015). Herbivore associated elicitor-induced defences are highly specific among closely related *Nicotiana* species. BMC Plant Biol..

[48] Lortzing T (2017). Transcriptomic responses of *Solanum dulcamara* to natural and simulated herbivory. Mol. Ecol. Resour..

[49] Steppuhn A, Gase K, Krock B, Halitschke R, Baldwin IT (2004). Nicotine’s defensive function in nature. PLoS. Biol..

[50] Wink M, Theile V (2002). Alkaloid tolerance in *Manduca sexta* and phylogenetically related sphingids (Lepidoptera: Sphingidae). Chemoecology.

[51] Hettenhausen C, Schuman MC, Wu JQ (2015). MAPK signaling: A key element in plant defense response to insects. Insect Sci..

[52] Heinrich M, Baldwin IT, Wu JQ (2011). Two mitogen-activated protein kinase kinases, MKK1 and MEK2, are involved in wounding- and specialist lepidopteran herbivore *Manduca sexta*-induced responses in *Nicotiana attenuata*. J. Exp. Bot..

[53] Goodspeed D, Chehab EW, Min-Venditti A, Braam J, Covington MF (2012). *Arabidopsis* synchronizes jasmonate-mediated defense with insect circadian behavior. Proc. Natl. Acad. Sci. USA.

[54] Kim SG, Yon F, Gaquerel E, Gulati J, Baldwin IT (2011). Tissue specific diurnal rhythms of metabolites and their regulation during herbivore attack in a native tobacco. Nicotiana attenuata. PLoS. ONE.

[55] Stam JM (2014). Plant Interactions with Multiple Insect Herbivores: From Community to Genes. Annu. Rev. Plant Biol..

[56] Hilker M, Fatouros NE (2015). Plant responses to insect egg deposition. Annu. Rev. Entomol..

[57] Trauer U, Hilker M (2013). Parental legacy in insects: variation of transgenerational immune priming during offspring development. PLoS. ONE.

[58] Bandoly M, Steppuhn A (2016). Bioassays to investigate the effects of insect oviposition on a plant’s resistance to herbivores. BioProtocol.

[59] Nguyen D (2016). Drought and flooding have distinct effects on herbivore-induced responses and resistance in *Solanum dulcamara*. Plant, Cell & Environ..

[60] Gulati J, Kim S-G, Baldwin IT, Gaquerel E (2013). Deciphering herbivory-induced gene-to-metabolite dynamics in *Nicotiana attenuata* tissues using a multifactorial approach. Plant Physiol..

[61] Ruijter, J. M. *et al*. Amplification efficiency: linking baseline and bias in the analysis of quantitative PCR data. *Nucleic Acids Research* **37**, 10.1093/nar/gkp045 (2009). 10.1093/nar/gkp045 PMC2665230 19237396

[62] Ritchie ME (2015). Limma powers differential expression analyses for RNA-sequencing and microarray studies. Nucleic Acids Research.

[63] Ritchie ME (2007). A comparison of background correction methods for two-colour microarrays. Bioinformatics.

[64] Bolstad BM, Irizarry RA, Astrand M, Speed TP (2003). A comparison of normalization methods for high density oligonucleotide array data based on variance and bias. Bioinformatics.

[65] Wickham, H. *ggplot2 Elegant graphics for data analysis* (Springer, 2009).

