# Supplementary material for: Moth oviposition shapes the species-specific transcriptional and phytohormonal response of Nicotiana attenuata to larval feeding
Source: Sci Rep. 2018 Jul 6;8:10249. doi: 10.1038/s41598-018-28233-z (PMC6035172; doi:10.1038/s41598-018-28233-z)
Supplement: Supplementary file 1 — Supplementary Information [file 41598_2018_28233_MOESM1_ESM.pdf]

Supplementary Information

Title of the study: "Moth oviposition shapes the species-specific transcriptional and phytohormonal response of *Nicotiana attenuata* to larval feeding"

Authors: Drok, S., Bandoly, M., Stelzer, S., Lortzing, T., Steppuhn, A.

Content

Supplementary Figures

Figure S1. Levels of phytohormones in *N. attenuata* in response to oviposition and/or feeding by lepidopteran larvae.

Figure S2. Numbers of genes regulated in *N. attenuata* plants by feeding and oviposition.

Figure S3. Correlations of the transcriptomic changes in response to feeding by either of two moth species with those in the cross-species treatments.

Figure S4. Expression patterns of genes analysed by microarray analysis and by qPCR.

Figure S5. Expression patterns of genes analysed by microarray analysis and by qPCR (including all feeding-induced treatments).

Supplementary Tables

Table S1. Genes that are significantly regulated in response to oviposition by either of two moth species in systemic leaves of *N. attenuata*.

Table S2. Effects of oviposition and feeding by either of two moth species on phytohormone levels of *N. attenuata.*

Table S3. Effects of oviposition and feeding by either of two moth species on gene expression quantified by qPCR

Table S4. Differences between all treatments involving larval feeding in gene expression quantified by qPCR.

Table S5. Annotation and primer information for the genes selected for qPCR analysis.

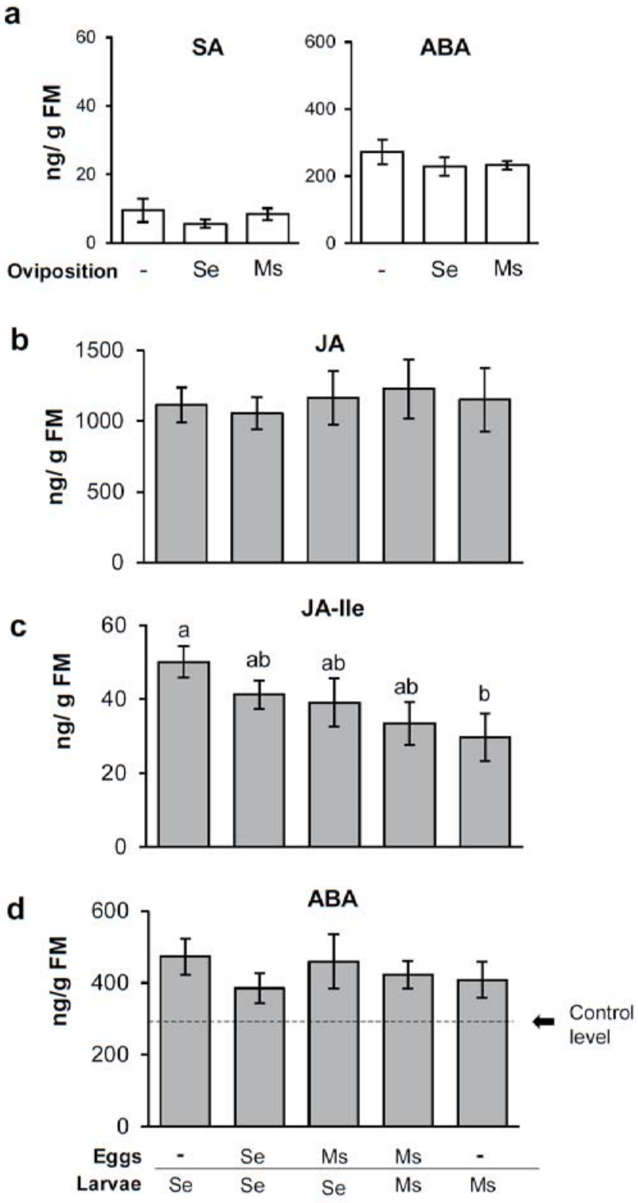

Figure S1. Levels of phytohormones in *N. attenuata* in response to oviposition and/or feeding by lepidopteran larvae. Mean content ± SEM of **(a)** salicylic acid (SA) and abscisic acid (ABA) in plants without (-) or with prior oviposition by *S. exigua* (Se) or *M. sexta* (Ms) at a lower leaf. Content of **(b)** jasmonic acid (JA), **(c)** the JA-conjugate with isoleucine (JA-Ile) and **(d)** ABA in *N. attenuata* leaves that were *S. exigua*- or *M. sexta*-fed for 24 hours. The plants were previously oviposited by either of the two species or not. An arrow demarks the ABA level of untreated control plants, which had JA and JA-Ile levels below the limit of quantification. All significant differences according to Kruskal Wallis analysis and pairwise Wilcoxon tests ($P < 0.05$) are marked by different letters.

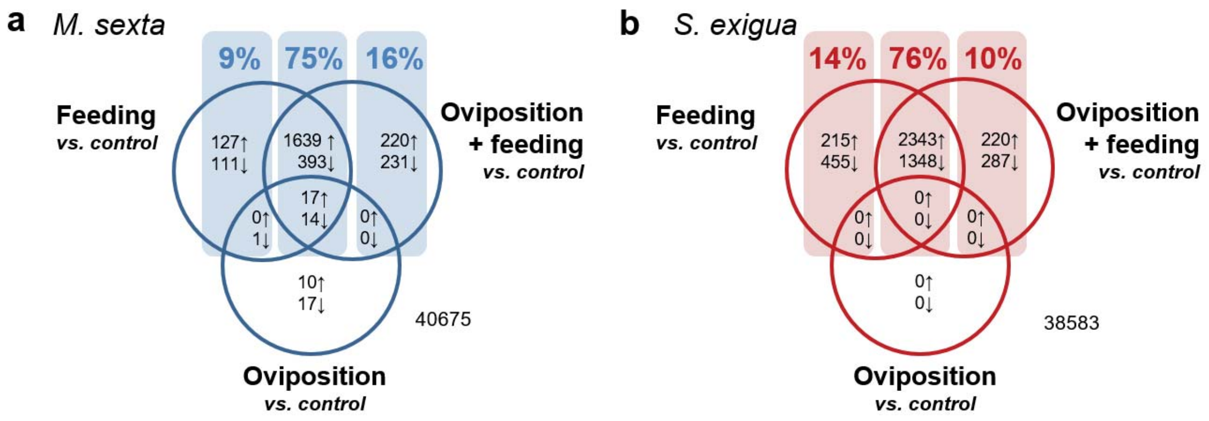

Figure S2. Numbers of genes regulated in *N. attenuata* plants by feeding and oviposition. Venn diagrams display the numbers of genes that were significantly up(↑) and down(↓)-regulated by oviposition and larval feeding of either **(a)** *M. sexta* or **(b)** *S. exigua* larvae (relative to untreated control plants) at $P < 0.05$ after correction for false discovery rate and with a $\log_2$-fold change (FC) > 1 in the microarray analysis.

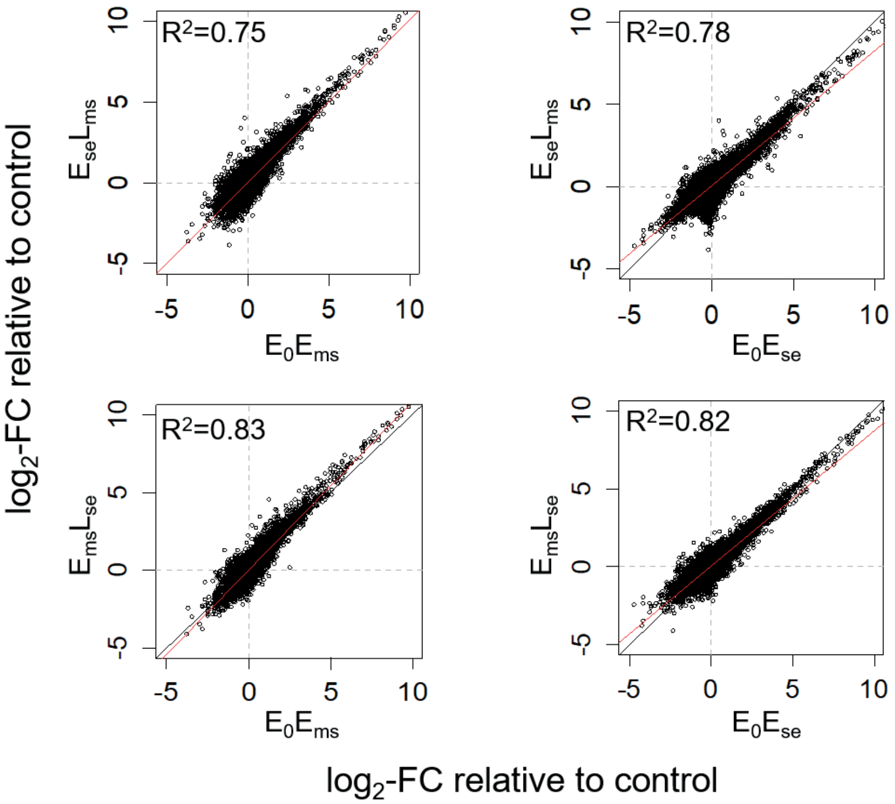

Figure S3. Correlations of the transcriptomic changes in response to feeding by either of two moth species with those in the cross-species treatments. Scatter plots of the $\log_2$-fold change (FC) in the transcript expression in pairwise comparisons of feeding-induced *N. attenuata* plants. Red lines signify the linear regression at the given coefficient of determination (R^2).

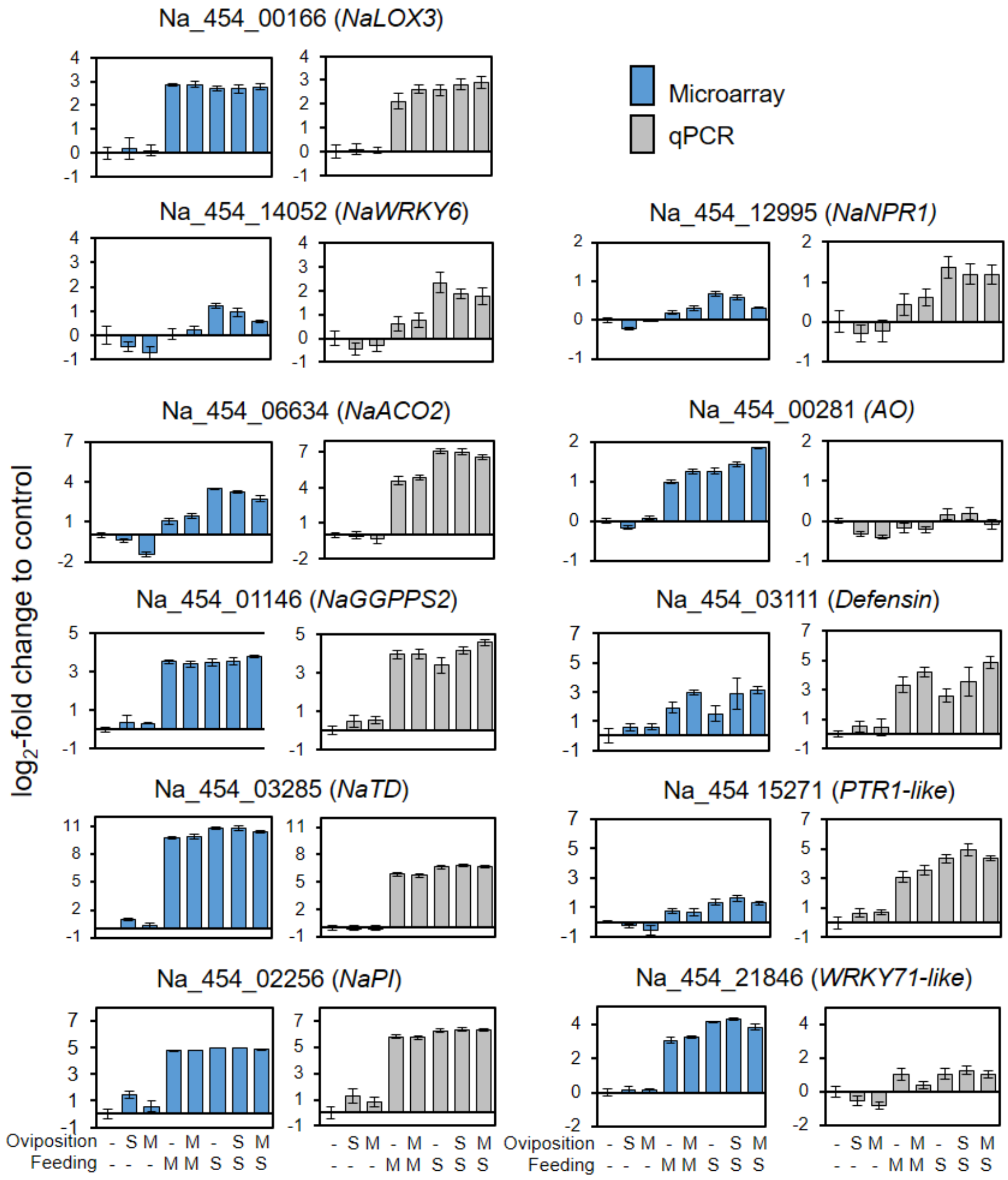

Figure S4. Expression patterns of genes analysed by microarray (blue bars, N = 3) and by qPCR (N = 9-10, normalized to *β-actin* and *EF1α* as reference genes). Transcript accumulation (mean ± SEM) displayed as $\log_2$-fold change relative to untreated control plants (--) in leaves of plants previously oviposited by *S. exigua* (S) or *M. sexta* (M) and non-oviposited (-) plants that were exposed to larval feeding or not. Statistics are provided in the corresponding main figures (Fig. 5 and Fig. 6) and in Table S3.

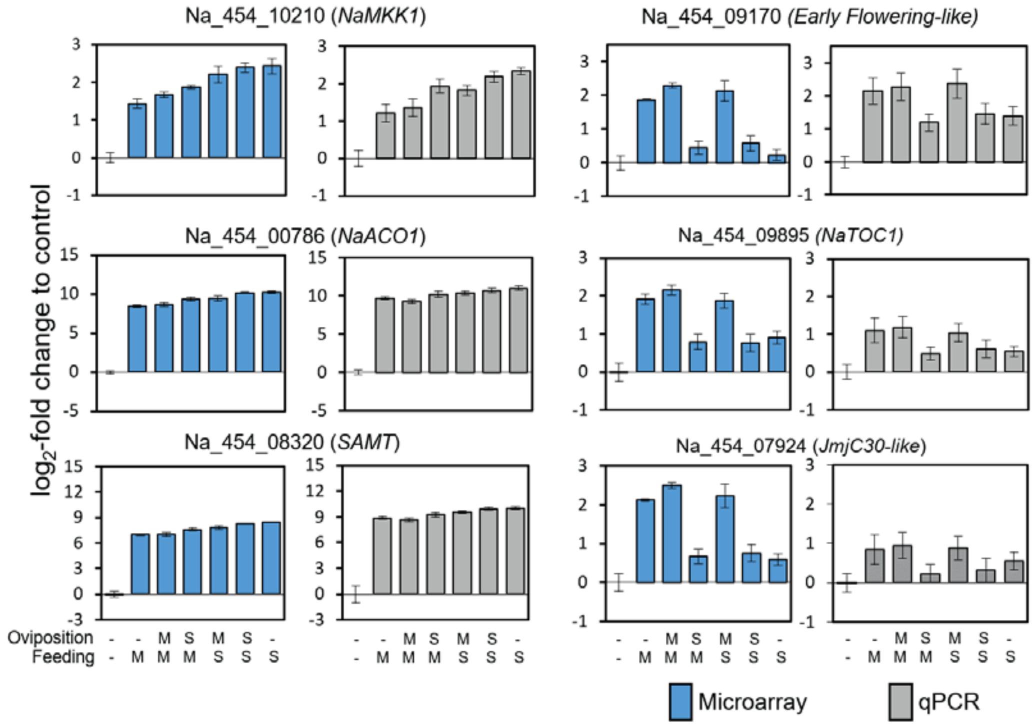

Figure S5. Expression patterns of genes analysed by microarray (blue bars, N = 3) and by qPCR (N = 9-10, normalized to *β-actin* and *EF1α* as reference genes). Transcript accumulation (mean ± SEM) displayed as log_2-fold change relative to untreated control plants (--) in leaves of plants previously oviposited by *S. exigua* (S) or *M. sexta* (M) and non-oviposited (-) plants that were exposed to larval feeding or not. Statistics are provided in Table S4.

Table S1: Genes that are significantly regulated in response to oviposition by *M. sexta* in systemic leaves of *N. attenuata*. Asterisks (*/**) indicate significant differences at $P < 0.05/0.01$ according to linear models and corrected for false discovery rate after Benjamini-Hochberg (N = 3 pools of 3 - 4 plants).

Class	Oligo ID	Log_2-FC	*P*-value	Description
**Amino acid metabolism**	Na_454_29928	-3.56	*	subtilisin-like protease SBT2.5
	Na_454_02188	-1.54	*	kunitz-type inhibitor d
	Na_454_12786	-1.01	*	14 kda proline-rich
	Na_454_04357	1	*	miraculin-like
	Na_454_21019	1.05	*	non-specific lipid-transfer protein 2-like
**Auxin-induced**	Na_454_13992	1.06	*	auxin-induced protein 15a-like
	Na_454_15320	1.13	*	auxin-induced protein 15a-like
**Cell wall modification**	Na_454_07546	-1.61	*	protein asc1-like
	Na_454_08203	1.53	**	expansin precursor
	Na_454_11643	-1.38	*	expansin-like b1
**Cytochrome P450**	Na_454_17662	-1.44	*	premnaspirodiene oxygenase-like
	Na_454_02033	-1.33	*	*Nicotiana tabacum* elicitor-inducible cytochrome P450 (CYP71D20)
	Na_454_24013	-1.05	*	premnaspirodiene oxygenase-like
**Development unspecified**	Na_454_13453	1.29	*	probable transporter mch1
	Na_454_15927	1.07	*	probable transporter mch1
	Na_454_23210	-1.14	*	phytosulfokines 3-like
**Ethylene signal transduction**	Na_454_09841	-1.55	**	ethylene-responsive transcription factor 1
	Na_454_06634	-1.45	**	*N. attenuata* 1-aminocyclopropane-1-carboxylate oxidase (NaACO2b)
	Na_454_26191	-1.2	*	ethylene response factor 4
**Regulation of transcription**	Na_454_13961	-1.44	**	probable serine threonine-protein kinase at1g18390
	Na_454_01573	-1.19	*	casp-like protein pimp1
	Na_454_20188	1.44	*	*N. attenuata* jasmonate ZIM domain protein f
	Na_454_17026	1.53	**	glycine rich protein-interacting protein
	Na_454_16844	1.67	**	*N. attenuata* timing ofcab expression 1/pseudo-response regulator 1 (TOC1/PRR1)
	Na_454_15118	1.83	*	protein early flowering 4-like
	Na_454_09895	2.14	**	*N. attenuata* timing of cab expression 1/pseudo-response regulator 1 (TOC1/PRR1)
	Na_454_07924	2.55	**	PREDICTED: *N. attenuata* lysine-specific demethylase JMJ30 (LOC109220083)
**Other**	Na_454_12179	1.21	**	probable starch synthase chloroplastic amyloplastic isoform x1
	Na_454_25391	-2.24	*	glutathione s-transferase u10-like
	Na_454_19395	1.36	*	PREDICTED: *Nicotiana tomentosiformis* pathogenesis-related protein PR-1 (LOC104101123)
	Na_454_08441	-1.63	**	protein plant cadmium resistance 2-like
	Na_454_14188	-1.37	**	hypothetical protein POPTR_0018s05900g
	Na_454_11566	-1.09	*	upf0392 protein rcom_0530710-like
	Na_454_17107	1.02	*	GDSL esterase/lipase
**Not assigned**	Na_454_10847	-1.1	*	*Nicotiana tomentosiformis* uncharacterized LOC104114715 (LOC104114715)
	Na_454_19303	-1.32	*	PREDICTED: Nicotiana sylvestris putative uncharacterized protein DDB G0292636 (LOC104222448)
	Na_454_02938	1.19	*	PREDICTED: uncharacterized protein LOC104109237
	Na_454_19036	1.56	*	PREDICTED: uncharacterized protein LOC104113476 isoform X1
	Na_454_09001	1.46	*	PREDICTED: uncharacterized protein LOC104245511
	Na_454_33970	1.2	*	PREDICTED: uncharacterized protein LOC104245511
	Na_454_21980	1.19	*	PREDICTED: uncharacterized protein LOC104225873 isoform X2
	Na_454_13724	1.16	*	PREDICTED: uncharacterized protein LOC104114536
	Na_454_04191	-1.02	*	PREDICTED: uncharacterized protein LOC104243756
	Na_454_23235	-1.04	*	PREDICTED: uncharacterized protein LOC104095139
	Na_454_15406	-1.12	*	PREDICTED: uncharacterized LOC104236994 (LOC104236994)
	Na_454_26475	-1.14	*	PREDICTED: uncharacterized protein LOC104216739
	Na_454_17182	-1.22	*	PREDICTED: uncharacterized protein LOC104229087
	Na_454_14832	-2.22	**	PREDICTED: uncharacterized protein LOC104240675

Table S1 continued

Class	Oligo ID	Log_2-FC	*P*-value	Description
**Not assigned**	Na_454_09642	1.06	**	Not assigned
	Na_454_38350	1.32	**	Not assigned
	Na_454_38683	1.18	*	Not assigned
	Na_454_15404	1.15	*	Not assigned
	Na_454_34315	1.12	*	Not assigned
	Na_454_26831	-1.5	*	Not assigned
	Na_454_11392	-1.94	*	Not assigned
	Na_454_22052	-1.98	**	Not assigned
	Na_454_12337	-2.04	*	Not assigned
	Na_454_34486	-2.14	*	Not assigned
	Na_454_43165	-2.52	*	Not assigned

Table S2: Effects of oviposition and feeding by either of two moth species on phytohormone levels of *N. attenuata*. In separate LMMs for each species (*S. exigua* or *M. sexta*) we tested for effects of the factors oviposition and herbivory and their interaction on *N. attenuata*'s phytohormone levels. The replicate blocks were included as random factor. Significant differences are emphasised in bold and reveal a significant effect of herbivory but no effect of the oviposition on all assessed phytohormones (N = 9 - 11).

		S. exigua			***M. sexta***		
		**Eggs**	**Feeding**	**Eggs*feeding**	**Eggs**	**Feeding**	**Eggs*feeding**
**SA**	*F*-value	0.3	89	0.4	1.2	17.2	2.4
**ng / g FM**	*P*-value	0.4206	**< 0.001**	0.5384	0.06	**< 0.001**	0.123
**ABA**	*F*-value	2.7	32	0.1	0.1	25.6	0.8
**ng / g FM**	*P*-value	0.359	**< 0.001**	0.713	0.682	**< 0.001**	0.361
**JA**	*F*-value	0.1	186.6	0.1	0.5	74.8	0.5
**ng / g FM**	*P*-value	0.998	**< 0.001**	0.725	0.331	**< 0.001**	0.492
**JA-Ile**	*F*-value	2.1	265.8	2.1	0.7	64.8	0.7
**ng / g FM**	*P*-value	0.999	**< 0.006**	0.147	0.224	**< 0.001**	0.389

Table S3: Effects of oviposition and feeding by either of two moth species on gene expression quantified by qPCR. We evaluated the effects of feeding and oviposition by either *S. exigua* and *M. sexta* on transcript accumulation of *N. attenuata genes* using a LMM. The replicate blocks were included as random factor. Significant differences are emphasised in bold (N = 9 - 11). Additionally, we tested whether feeding by both species elicited different gene expression levels using Welch's *t* -test (right columns). Log_2-fold changes in gene expression relative to control plants are presented in Fig. 5, 6 and Fig. S4.

		M. sexta			***S. exigua***			***M.sexta* feeding vs. *S.exigua* feeding**	
		**Eggs**	**Feeding**	**Eggs* feeding**	**Eggs**	**Feeding**	**Eggs* feeding**		
**Na_454_00166 (Na_LOX3)**	*F* -value	1.38	129.14	2.63	1.83	292.51	1.19	*t*	-1.23
	df	1	1	1	1	1	1	df	16
	P -value	0.240	**< 0.001**	0.105	0.177	**< 0.001**	0.276	*P* -value	0.237
**Na_454_00281 (AO)**	*F* -value	5.73	0.24	7.92	0.51	11.36	4.01	*t*	-1.81
	df	1	1	1	1	1	1	df	18
	P -value	**0.017**	0.623	**0.005**	0.474	**< 0.001**	**0.045**	*P* -value	0.088
**Na_454_01146 (NaGGPPS2)**	*F* -value	4.72	558.34	2.15	14.42	253.83	0.54	*t*	1.60
	df	1	1	1	1	1	1	df	12
	P -value	**0.030**	**< 0.001**	0.143	**0.001**	**< 0.001**	0.463	*P* -value	0.138
**Na_454_02256 (NaPI)**	F-value	3.89	301.35	1.18	5.44	335.65	3.00	*t*	-2.11
	df	1	1	1	1	1	1	df	18
	P -value	0.314	**< 0.001**	0.278	**0.020**	**< 0.001**	0.083	*P* -value	**0.049**
**Na_454_03111 (Defensin)**	*F* -value	4.31	91.40	0.77	3.56	47.99	0.63	*t*	1.07
	df	1	1	1	1	1	1	df	16
	P -value	**0.0378**	**< 0.001**	0.3812	0.0687	**< 0.001**	0.4267	*P* -value	0.299
**Na_454_03285 (NaTD)**	F-value	0.33	1575.45	0.00	1.30	2442.05	2.07	*t*	-3.39
	df	1	1	1	1	1	1	df	18
	P -value	0.564	**< 0.001**	0.958	0.254	**< 0.001**	0.150	*P* -value	**0.003**
**Na_454_06634 (NaACO2)**	*F* -value	0.02	331.69	1.94	0.04	1333.01	0.07	*t*	-6.03
	df	1	1	1	1	1	1	df	13
	P -value	0.874	**< 0.001**	0.164	0.842	**< 0.001**	0.790	*P* -value	**< 0.001**
**Na_454_12995 (NaNPR1)**	F-value	0.02	32.59	2.78	0.03	125.27	2.63	*t*	-3.18
	df	1	1	1	1	1	1	df	15
	P -value	0.754	**< 0.001**	0.095	0.797	**< 0.001**	0.105	*P* -value	**0.006**
**Na_454_14052 (Na_WRKY6)**	*F* -value	0.01	8.60	0.94	0.73	106.87	0.70	*t*	-5.43
	df	1	1	1	1	1	1	df	16
	P -value	0.874	**0.003**	0.332	0.394	**< 0.001**	0.402	*P* -value	**< 0.001**
**Na_454_15272 (PRT1)**	*F* -value	5.04	129.99	0.02	8.30	271.31	0.00	*t*	-2.98
	df	1	1	1	1	1	1	df	18
	P -value	**0.025**	**< 0.001**	0.886	**0.004**	**< 0.001**	0.960	*P* -value	**0.008**
**Na_454_21846 (WRKY71-like)**	*F* -value	12.61	26.93	0.40	0.42	65.76	6.60	*t*	-0.46
	df	1	1	1	1	1	1	df	17
	P -value	**< 0.001**	**< 0.001**	0.5289	0.5162	**< 0.001**	**0.0102**	*P* -value	**0.9641**

Table S4: Differences between all treatments involving larval feeding in gene expression quantified by qPCR. We evaluated the effects of feeding and prior oviposition treatments on transcript accumulation of *N. attenuata genes* using a LMM. The replicate blocks were included as random factor. Significant differences are emphasised in bold (N = 9-11). Tukey tests were used to verify differences between the individual treatments. Log_2-fold changes in gene expression relative to control plants are presented in Fig.7 and Fig. S5.

			SAMT		***NaMKK1***		***NaACO***		***early flowering-like***		***NaTOC1***		***JmjC30-like***	
			F -value	*P* -value	*F* -value	*P* -value	*F* -value	*P* -value	*F* -value	*P* -value	*F* -value	*P* -value	*F* -value	*P* -value
			9.53	**<0.001**	7.82	**<0.001**	9.14	**<0.001**	3.74	**0.002**	4.84	**<0.001**	5.11	**<0.001**
			Tukey contrasts											
			z -value	*P* -value	*z* -value	*P* -value	*z* -value	*P* -value	*z* -value	*P* -value	*z* -value	*P* -value	*z* -value	*P* -value
E_0 L_{ms}		E_0 L_{se}	4.42	**<0.001**	4.96	**<0.001**	4.35	**<0.001**	-1.74	0.507	-2.65	0.086	-2.91	**0.042**
E_0 L_{ms}		E_{se} L_{se}	4.02	**<0.001**	4.33	**<0.001**	3.35	**0.010**	-2.35	0.175	-2.08	0.299	-2.71	0.074
E_0 L_{ms}		E_{ms} L_{se}	-2.70	0.076	-2.70	0.075	-2.14	0.269	-0.26	1.000	-0.66	0.986	0.33	1.000
E_0 L_{ms}		E_{se} L_{ms}	1.44	0.703	3.20	**0.017**	1.09	0.885	-2.88	**0.046**	-2.75	0.065	-3.79	**0.002**
E_0 L_{ms}		E_{ms} L_{ms}	1.03	0.910	-0.66	0.986	1.43	0.707	-0.03	1.000	-0.32	1.000	0.56	0.993
E_0 L_{se}		E_{se} L_{se}	0.26	1.000	0.47	0.997	0.85	0.957	0.60	0.991	-0.62	0.990	-0.20	1.000
E_0 L_{se}		E_{ms} L_{se}	1.72	0.518	2.26	0.209	2.22	0.231	-1.99	0.347	-3.27	**0.014**	-2.59	0.100
E_0 L_{se}	*ver-sus*	E_{se} L_{ms}	2.98	**0.034**	1.76	0.493	3.11	**0.023**	1.07	0.894	-0.04	1.000	0.78	0.970
E_0 L_{se}		E_{ms} L_{ms}	5.45	**<0.001**	4.30	**<0.001**	5.78	**<0.001**	-1.72	0.518	-2.89	**0.045**	-2.29	0.199
E_{se} L_{ms}		E_{ms} L_{ms}	2.46	0.135	2.54	0.112	2.47	0.132	-2.83	0.052	-3.01	**0.032**	-3.13	**0.022**
E_{ms} L_{se}		E_{se} L_{se}	1.41	0.723	1.72	0.519	1.29	0.792	-2.60	0.096	-2.72	0.071	-2.39	0.160
E_{se} L_{ms}		E_{se} L_{se}	2.63	0.091	1.23	0.821	2.19	0.245	0.46	0.998	0.60	0.991	0.99	0.923
E_{ms} L_{se}		E_{ms} L_{ms}	3.72	**0.003**	2.04	0.321	3.57	**0.005**	0.23	1.000	0.32	1.000	0.24	1.000
E_{ms} L_{se}		E_{se} L_{ms}	-1.26	0.807	0.51	0.996	-0.97	0.927	-3.14	**0.021**	-3.42	**0.008**	-3.46	**0.007**
E_{ms} L_{ms}		E_{se} L_{se}	5.01	**<0.001**	3.69	**0.003**	4.73	**<0.001**	-2.32	0.187	-2.34	0.179	-2.09	0.292

Table S5: Annotation and primer information for all genes selected for qPCR analysis. Sequences of the forward (F) and reversed (R) primers used and E-values of 'predicted' gene annotations according to blastx against the NCBI database of non-redundant protein sequences (nr). If available, we used primer sequences that were already used in previous studies. Otherwise we designed primers using the NCBI primer-design tool based on either published sequences of annotated genes or, if not available, on the p454 sequences of BioProject PRJNA223344.

Oligo ID	Gene	Description / E-value		Primer sequence (5'-3')
Na_454_00166	NaLOX3	*N. attenuata* lipoxygenase	F	GGCAGTGAAATTCAAAGTAAGAG
			R	CCCAAAATTTGAATCCACAACA
Na_454_00281	AO	Predicted L-aspartate oxidase *Nicotiana sylvestris*, E-value 0.0	F	ACATGGAACTGTGGCTGTGA
			R	TGAATCCAAAGGGCAGAGCA
Na_454_00283	NaActin	*N. attenuata* β-actin	F	CCGGTATTGTGTTGGACTCTGGTG
			R	CAGCTGAGGTGGTGAACATGTAACC
Na_454_00786	NaACO1	*N. attenuata* ACC oxidase ACO1	F	GCAACTGCATAGATCCAAATTCA
			R	TGTAGTAGGGACACACGCTT
Na_454_01146	NaGGPPS2	*N. attenuata* geranylgeranyl pyrophosphate synthase	F	CTGCTGGGATTGGAAAAGGC
			R	ACACCGTATAGCTCGAAGAA
Na_454_01437	NaEF1a	*N. attenuata* elongation factor 1a	F	CCACACTTCCCACATTGCTG
			R	CGCATGTCCCTCACAGCAAA
Na_454_02256	NaTPI	*N. attenuata* 7-domain trypsin inhibitor precursor	F	CTTTGTCTTGCCTGTAATCTGT
			R	TGCAGCAAAAGCATCACTAACT
Na_454_03111	DEF2	*N. alata* NaD1, E-value 2e-51 and *N. attenuata* DEF2 (Cloning vector pSOL9DEF2) E-value 5e-52	F	TTTGTTGCCTATGAGGTGCAAG
			R	TCAAACACACATGGCTTAGTGC
Na_454_03285	NaTD	*N. attenuata* threonine deaminase	F	TCTCTGTCAGTGGAAGCTAGT
			R	TCCTACTAAAGGTTCTCGGT
Na_454_06634	NaACO2	*N. attenuata* ACC oxidase ACO2b	F	TTCTGAGAGAGCTGACACCAT
			R	CATGCACTTCTTGTAATGTCCCT
Na_454_07924	Jmjc30	Predicted lysine-specific demethylase JMJ30 (*N. attenuata*)	F	CACTATGTCCACGTCAGCCA
			R	TATCCGCCTTCCTCCGCTAT
Na_454_08320	SAMT	Predicted salicylate carboxymethyltransferase-like (*N. attenuata*) E-value 1e-176	F	AGCACGGGTTTCAGTCACCA
			R	AGTGCAAGCTGTTGGAAGGG
Na_454_09170	Early flowering 4-...	Predicted Early flowering 4-like protein (*N. attenuata)* E-value 2e-90	F	GAGGTGCAGTCGGTGTTGGA
			R	ACGCCGTTCTTCCGCTGGTAA
Na_454_09895	NaTOC1	*N. attenuata TIMING OF CAB EXPRESSION 1/PSEUDO-RESPONSE REGULATOR 1 (TOC1/PRR1)*	F	TGGTCAACATGCCCACAAATG
			R	CCCTGTCCAGATTGCTTCTCT
Na_454_10210	NaMKK1	*N. attenuata* MKK1	F	ATCCCGAGATTCGCCGTCAA
			R	TCGAGTGTGCCGGAGTTCAT
Na_454_12995	NaNPR1	*N. attenuata* NPR1	F	AGCCCAATAAGCTCCCTTTT
			R	ACAAGTGTTGTATGTATGTTGAC
Na_454_14052	NaWRKY6	*N. attenuata* WRKY6	F	TCGATGTAATTTATGATAGTTTTTGC
			R	CAAATATCTTAATGGGGAATTACAGC
Na_454_15271	NTR1/PTR	Predicted protein NRT1/ PTR FAMILY 6.3-like (*Nicotiana tomentosiformis*), E-value 2e-71	F	TGGGCTAGTGGTGCAATGAT
			R	TGGCTGAAGAAGCATTCCCT
Na_454_21846	WRKY 71	Predicted WRKY transcription factor 71 (*N. sylvestris*), E-value 5e-76	F	TGGCTCATTTCACGCAATCAA
			R	ACGAAGAGTTGCGGGACAAT
Na_454_29412	PLX2	Predicted leucine-rich repeat receptor-like protein kinase PXL2 (*Nicotiana tomentosiformis*) E-value 3e-121	F	ACTCCAAAGGTGCAGTTGAGA
			R	TGCTGACATTCCAAGACCCA
